# BMP4/LIF or RA/Forskolin Suppresses the Proliferation of Neural Stem Cells Derived from Adult Monkey Brain

**DOI:** 10.1155/2017/7012405

**Published:** 2017-09-20

**Authors:** Xinxin Han, Liming Yu, Qingqing Chen, Min Wang, Jie Ren, Guangming Wang, Yihong Chen, Lixia Lu, Haibin Tian, Li Chen, Ying Zhang, Yuehua Liu, Hua He, Zhengliang Gao

**Affiliations:** ^1^Shanghai Stomatological Hospital, Fudan University, Shanghai 200001, China; ^2^School of Medicine, Jiaxing University, Zhejiang 314001, China; ^3^Shanghai Tenth People's Hospital, Tongji University School of Medicine, Shanghai 200092, China; ^4^Changzheng Hospital, Second Hospital Affiliated with Second Military Medical University, Shanghai 200003, China; ^5^Tongji University Advanced Institute of Translational Medicine, Shanghai 200092, China

## Abstract

Monkeys are much closer to human and are the most common nonhuman primates which are used in biomedical studies. Neural progenitor cells can originate from the hippocampus of adult monkeys. Despite a few reports, the detailed properties of monkey neural stem cells (NSCs) and their responses to cytokine are still unclear. Here, we derive NSCs from an adult monkey brain and demonstrate that BMP4 inhibits cell proliferation and affects cell morphology of monkey NSCs. Combined treatment of BMP4 and LIF or RA and Forskolin represses the proliferation of monkey NSCs. We also show that BMP4 may promote monkey NSC quiescence. Our study therefore provides implications for NSC-based cell therapy of brain injury in the future.

## 1. Introduction

An adult mammalian brain shows amazing plasticity by regenerating new neural cells after injury or damage [1, 2]. In the brain, neural regeneration mainly arises from the differentiation of endogenous neural stem cells (NSCs), which exists in subventricular zone (SVZ) and subgranular zone (SGZ). SVZ is in lateral ventricle, and SGZ is in the dentate gyrus (DG) of the mammalian brain [3]. The DG area in hippocampus constantly produces new cells throughout the life. Newborn neuron cells are activated to support the memory and cognition particularly in their plasticity phase [4].

Stem cells possess the ability to self-renew and differentiate into diverse progeny cells [2, 5]. NSCs belong to multipotent cells and can differentiate into neurons, astrocytes, and oligodendrocytes [6, 7].

Neural regeneration always requires neuron protection and axon regeneration [8]. NSCs are responsible for brain plasticity and repair by producing, restoring, and modifying central nervous system (CNS) [9]. Due to the limited number of NSCs in CNS, one of the key strategies of brain repair is transplanting NSCs into CNS. Three decades ago, fetal tissue was grafted into Parkinson's patients for brain repair [10]. However, the wide application of fetal tissue transplantation is hampered by various ethical issues [11]. Induced pluripotent stem cells (iPSCs) have been considered as a new approach for cell therapy [12, 13]. Tissue damage provides critical signals for cellular reprogramming [14]. Fibroblast and astroglial cells also have been transdifferentiated into neurons for CNS repair [15, 16]. However, iPSC-based cell therapy also encounters problems such as low efficiency and safety issues.

One way of neural regeneration is utilizing endogenous NSCs to generate newborn neurons. Endogenous NSCs survive in stem cell niches which receive the support from microenvironments [17, 18]. When damage or disease (such as stroke) occurs, NSCs' proliferation in adult brain niches increases and migrates to brain ischemic areas [3, 19, 20]. Newborn endogenous neurons can be recruited and integrated into local circuits [21]. However, the in vivo neurogenesis ability is restricted and only a few new neurons could be produced, which are inadequate for brain repair [22, 23].

NSCs can survive in the DG regions of hippocampus throughout an individual's life-span, but human VZ and SVZ regions stop to produce neurons at 2 years old [24]. Despite NSCs have long time activity in adult hippocampus, their amount decreases with age and significantly declines in Alzheimer's disease (AD) transgenic mouse [25]. This reduction of NSCs causes learning and memory loss [25]. It is vital that NSCs maintained the proliferating activity by the stem cell niche which are consisted by various cytokines [9]. For example, IGF (insulin-like growth factor), FGF (fibroblast growth factor), and Noggin (a BMP inhibitor, encoded by the NOG gene) increase NSC proliferation [26]. Dkk1 (Wnt antagonist Dickkopf-1) is increased along with aging, and loss function of Dkk1 can enhance neurogenesis in the hippocampus [27].

Mouse and rat often are used as a model organism for mammalian development research. Nonetheless, the growth mechanisms of mammals are different among species [28]. Monkeys, especially rhesus macaque, are the most universal nonhuman primates used in biomedical research, particularly for disease modeling which are special for advanced animals (such as HIV, poliomyelitis, and and aging) due to a close evolutionary and genomic relationship with humans [29, 30].

At present, most researches of monkey neural stem cells focus on embryonic stem cells differentiating into neural stem cells. There are very few studies on adult monkey neural stem cells. For example, monkey neural stem and progenitor cells can differentiate into immature oligodendrocytes [31]. Brain-derived neurotrophic factor (BDNF) promotes NPC proliferation and induces cynomolgus monkey neural progenitor differentiation into neurons [32]. Study on transplantation of adult monkey neural stem cells also showed that monkey NSCs can be injected into a contusion spinal cord injury model in rhesus macaque monkeys [33]. However, detailed cell properties of adult monkey NSCs and factors except BDNF that can regulate monkey NSC proliferation are still unknown.

In this study, we generated NSCs from monkey brain and investigated the proliferation ability. We found bone morphogenetic protein 4 (BMP4) inhibited monkey NSC proliferation and changed the morphology of monkey NSCs. Combined application of BMP4 and LIF (leukocyte inhibitor factor) or RA (retinoic acid) and Forskolin suppressed cell proliferation. We also examined the differentiation tendency under these cytokine treatments. These results may provide useful information for brain injury repair using stem cell-based therapy.

## 2. Materials and Methods

### 2.1. Experimental Monkey

Animals were fed according to the requirements of the Animal Welfare Act, and protocols were followed based on the permission implemented by the animal ethics committee of JOINN Laboratories (Suzhou). The animals were fed under conditions approved by the Association for the Assessment and Accreditation of Laboratory Animal Care International. A male rhesus monkey (*Macaca fascicularis*, 4 kg, 3 years old) was used for studies.

### 2.2. Derived NSCs from Monkey Brain

A monkey's brain was removed and collected in accordance with Regulations for the Administration of Affairs Concerning Experimental Animals. Brain tissue was washed in HBSS—Hank's buffer (Gibco) for 8 times. Then, the hippocampus and temporal cortex were dissected into the dishes. These selected tissues were crushed by a sterile scalpel and surgical scissors and then added 30 mL phosphate-buffered saline (0.01 M PBS, pH 7.2) to the tissues. Tissue suspension was passed through 70 *μ*m sieve, and filtered tissue liquid was collected to tubes. The tissue mixture was digested with 1 U/mL dispase II (Roche) at 37°C for 45 minutes and then was centrifuged at 1000*g* for 3 minutes. The centrifuged suspension was discarded, and a precipitate was washed by NSC growth medium (DMEM/F12/N2/B27/GlutaMAX/penicillin/streptomycin/ (20 ng/mL FGF)/(20 ng/mL EGF)/(20 ng/mL heparin)). Then, cell suspension mixture was centrifuged at 1000*g* for 3 minutes. The precipitate containing primary cells was resuspended and seeded into 100 mm dishes at different concentration in medium. Then, the medium was half refreshed every 2 days. After 2 months of culture, NSCs were obtained which originated from an adult monkey brain.

### 2.3. Neurosphere Formation and NSC Culture

After 2 months of careful culture, we first observed neurospheres originating from an adult monkey brain. Then, NSCs climbed out from the neurospheres. When the NSC clone was large, NSC clones were washed twice by using DMEM/F12 then added 2 mL dispase II (1 U/mL dissolved in DMEM/F12) to the NSC clones. Cell dishes were incubated at 37°C for 5 minutes then added 3 mL growth medium. Cells were collected into tubes and centrifuged at 1000*g* for 3 minutes. Then, cells were resuspended and planted to new 100 mm dishes at 1 : 4 ratios. NSCs were passaged and expanded depending on this protocol.

### 2.4. Factor Treatment

NSCs were passaged and cultured in growth medium containing lower FGF (5 ng/mL) for 12 hours. Then, the NSCs were treated with different factors in 6 groups as follows: (1) control; (2) BMP4 (100 *μ*g/mL); (3) BMP4 (100 *μ*g/mL) and LIF (leukocyte inhibitor factor, 50 ng/mL); (4) RA (retinoic acid, 1 *μ*M) and Forskolin (5 *μ*M); (5) 10% fetal bovine serum (FBS) in DMEM/F12/GlutaMAX/FGF/EGF/heparin/penicillin streptomycin; and (6) no factor growth medium (DMEM/F12/N2/B27/GlutaMAX/FGF/EGF/heparin/penicillin streptomycin). These NSCs were cultured at 37°C in 5% CO_2_ incubator for 6 days.

### 2.5. Antibodies and Immunostaining

For antibody staining, cells were fixed by using 4% PFA and washed with PBS for 3 times. Then, they were permeabilized with 2.5% Triton X-100 for 10 minutes. Cells were blocked with 5% BSA for 1 hour at room temperature. According to the general immunofluorescence procedure, cells were washed with 0.1% Tween-20 in PBS and incubated with primary antibody at 4°C for 48 hours. Primary antibodies used for immunostaining were Sox2 (R&D), Nestin (R&D), and Ki67 (Thermo Fisher). The dilution buffer of primary antibody was 2.5% BSA in PBS. Cells were washed with 0.1% Tween-20 in PBS for 3 times and incubated with a second antibody at room temperature for 1.5 hours. At last, we used 4′,6-diamidino-2-phenylindole (DAPI, Sigma) to mark the nucleus of monkey NSCs. Additional attention, when using DAPI, we must treat the cells for 10 minutes at room temperature after second antibody incubation.

### 2.6. Microscopic Image

Cells were observed by using an inverted fluorescence microscope (Nikon TE2000). Images were acquired under a color CCD camera and digitized by a PC-based frame grabber. Then, photos were analyzed and checked in ImageJ software. Data collected from ImageJ were calculated by Excel. Calculation results were analyzed by the GraphPad Prism 6 software and then were organized into charts.

### 2.7. Statistical Analysis

We manually drew the frames of neurosphere, NSC clones, or differentiation cells of NSCs based on morphology. Then, we recorded areas or lengths by ImageJ software. Meanwhile, we drew bar's area or lengths in the same image as a ruler. Compared with the bar, actual size was counted and recorded. All data were showed as mean ± standard deviation of the mean (SD). Data was calculated by Excel and *p* value was measured for statistical significance by two-tailed Student's *t*-test.

## 3. Results and Discussion

### 3.1. Results

#### 3.1.1. Improved Process and Cell Proliferation Rate of NSCs from an Adult Monkey

**Figure 1:**
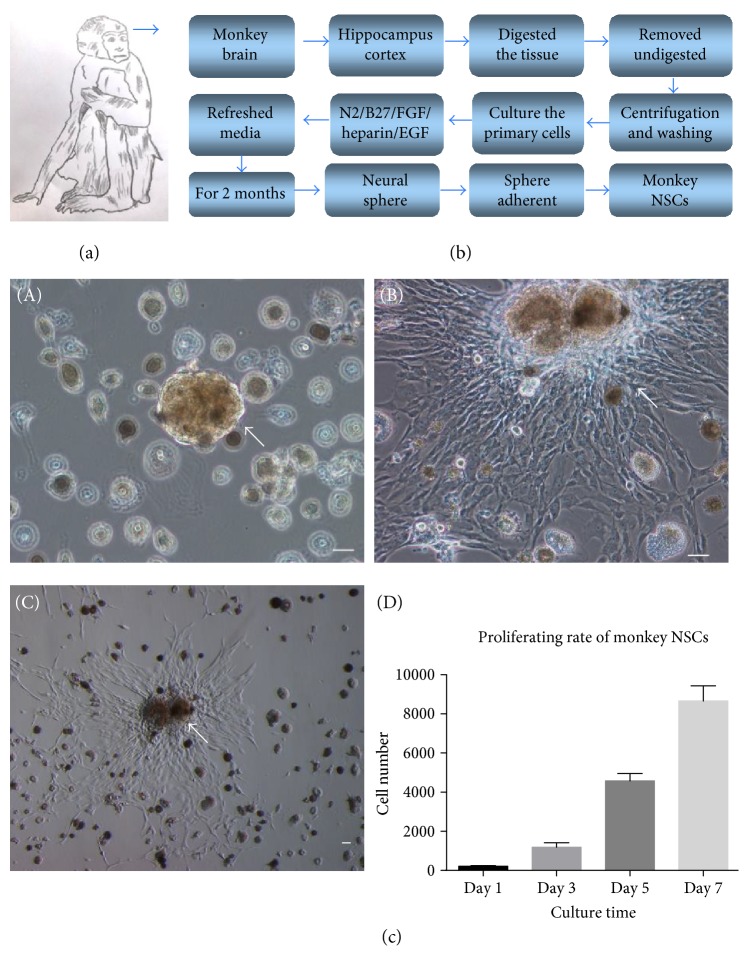
A flow chart of a method used in neural stem cell-like cell isolation from the monkey brain (*Macaca fascicularis*). (a) Primary cells were obtained from the cultured monkey brain. (b) The detail of cell-isolating process was described. The monkey brain cortex was treated with enzyme and cultured for two months. Within these months, fresh-specific medium was changed to neural stem cells (cell sedimentation after centrifugation) every two days. (c) Monkey neurospheres occurred, and neural stem cell-like cells proliferated from neurospheres. (A) Monkey neurospheres appeared in adult monkey brain culture medium. (B) Neural stem cell-like cells proliferated from neurospheres. (C) Large number of neural stem cell-like cells formed an independent clone, and this clone was seen at low-magnification microscope. (D) The proliferating rate of monkey NSC-like cells was shown. Scale bar = 50 *μ*m.

To obtain the development secret of NSCs in nonhuman primates, we designed a process of isolating monkey NSCs from hippocampus and temporal cortex of an adult monkey brain (Figure 1). The male adult monkey (*Macaca fascicularis*) (Figure 1(a)) was used as a donor. A flow chart of the method was designed for deriving neural stem cell-like cells from an adult monkey (Figure 1(b)). Cells originated from primary culture of the monkey brain. The detail of isolating process was shown. The hippocampus and cortex of the monkey brain were digested by enzyme and planted in different concentration for 2 months. During these 2 months, fresh medium was changed to cells every 2 days.

In order to test the proliferation status and rate of monkey NSC-like cells, we recorded and analyzed the proliferation of monkey neurospheres (Figure 1(c)). Monkey neurosphere appeared after 2 months of monkey brain culture (Figure 1(c), A). Neural stem cell-like cells were proliferated, and cells climbed out the neurosphere like waterfalls (Figure 1(c), B). Massive neural stem cell-like cells formed an independent clone around neurosphere (Figure 1(c), C). Monkey NSC-like cells proliferated rapidly based on the growth curve (Figure 1(c), D).

#### 3.1.2. Growth Process of Neural Stem Cell-Like Cells

**Figure 2:**
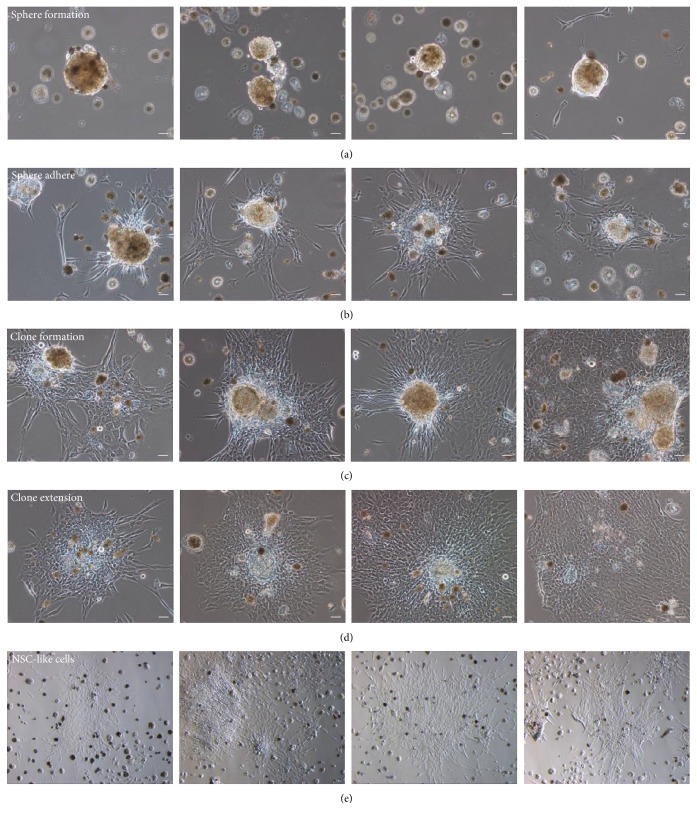
Growth process of neural stem cell-like cells derived from monkey. (a) Neurospheres formed in neural stem cell selective media with factors. (b) Spheres attached the plastic surface of the cell culture dish. (c) Cells climbed from the spheres and cell clone appeared. (d) Clone expanded the territory, and new cells are increased. (e) Monkey neural stem cell-like cells emerged. Scale bar = 50 *μ*m.

To examine the proliferation and development potential of cells derived from an adult, we fostered and observed these cells. Firstly, some visible cell balls appeared in dishes and these suspended floating balls (sphere) became bigger with time and culture process (Figure 2(a)). Subsequently, the suspended floating balls and spheres started to stick to the bottom of the dishes and sporadic cells climbed out from the adherent spheres (Figure 2(b)). Then, more and more cells emerged from adherent spheres and a stem cell clone was formed on the surface of the dish bottom (Figure 2(c)). As time went by, the clone extended quickly and cells which climbed out from the clone increased rapidly (Figure 2(d)). Finally, monkey neural stem cell-like cells emerged (Figure 2(e)).

#### 3.1.3. Proliferation and Division Potential Analysis of Neurospheres and NSCs

**Figure 3:**
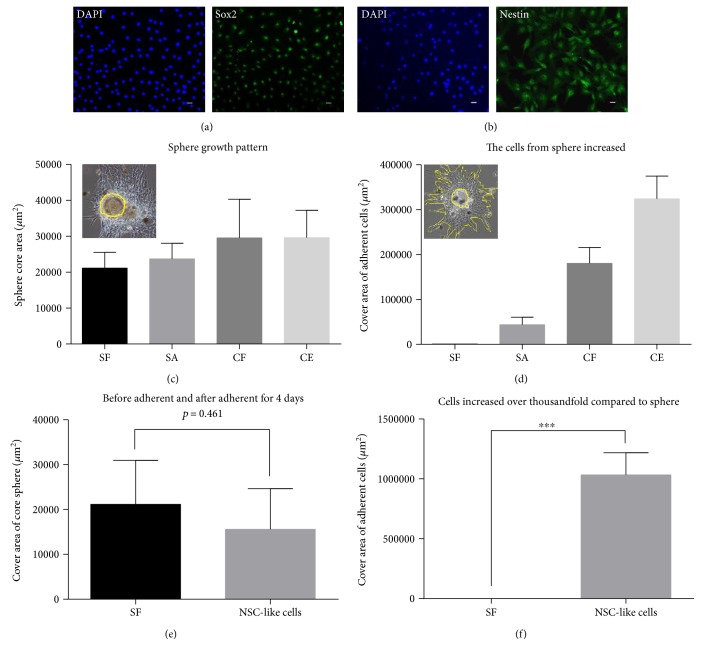
Characteristics of the neurospheres and neural stem cells. (a) Cells expressed neural stem cell marker protein Sox2. (b) Cells expressed neural stem cell maker protein Nestin. (c) Sphere growth pattern showed in yellow circles. Sphere core was slowly growing during the progress of sphere formation (SF), sphere adherence (SA), clone formation (CF), and clone extension (CE) in neural stem cell selective media with factors. (d) Cells proliferated from the spheres and adherent cell increased. Cells climbed from the spheres, and cover area of adherent cell pattern was showed in a big yellow border line. Yellow circles present the spheres. (e) Sphere size slightly seemed to be a downward trend but there was no significant difference. *p* value = 0.461. (f) Cell growth area from sphere was more than a thousand times versus sphere. Scale bar = 25 *μ*m. Data were shown as mean ± SD; ^∗∗∗^*p* value < 0.001.

To thoroughly investigate neural stem cells, we made use of neural stem cell marker protein antibodies for immune staining. SRY- (sex-determining region Y-) box 2, also known as Sox2, is a transcription factor that is essential for maintaining embryonic and neural stem cells. Nestin is a neuroectodermal stem cell marker and a type VI intermediate filament (IF) protein. We fixed NSC-like cells from the monkey brain and discovered these cells were Sox2 (Figure 3(a)) and Nestin (Figure 3(b)) positive. Cells expressing both Sox2 and Nestin were considered as the characteristics of NSCs.

To study the dynamic proliferation change and differentiation potential of neurospheres and NSCs, we calculated the sphere growth pattern as shown in yellow circles (Figure 3(c)). The core of the sphere grew slowly during the 4 sphere development progresses: sphere formation (SF), sphere adherence (SA), clone formation (CF), and clone extension (CE) (Figure 3(c)). We drew up cells area surging from spheres in a big yellow border line and measured cell's extending areas (Figure 3(d)). Yellow circles present spheres (Figure 3(d)). The data showed that cell's extending area was significantly increased through the 4 sphere development processes (Figure 3(d)). There was a slightly declining trend of sphere size but there were no significant differences (Figure 3(e)). Cells grew very quickly from the sphere, and the multiplication rate was more than a thousand times when compared with the initial stage (Figure 3(f)).

#### 3.1.4. BMP4 Inhibited Monkey NSC Proliferation and Affected Their Morphology

Bone morphogenetic proteins (BMPs) are members of transforming growth factor-*β* (TGF-*β*) super family [34]. BMP4 and BMP type I receptor (BMPRIA) are overexpressed in coronal phase of a rat molar. BMP4 can rescue the absence of molar germ which is caused by homeobox-containing transcription factor 1 (Msx1) knockout [35]. BMP also plays a major role in the formation and maintenance of a variety of tissues, such as induction of osteogenesis, cartilage, kidney, muscle, and fat [34].

**Figure 4:**
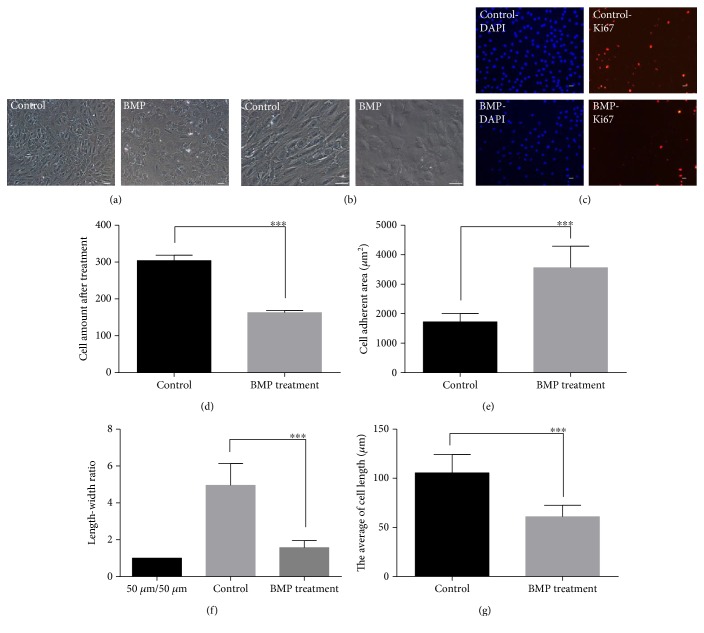
BMP4 inhibited cell proliferation and effected cell morphology. (a) Monkey NSCs treated in control media or BMP4 media for 6 days. (b) Cell morphology displayed the different phenotypes after BMP4 treatment under enlarged view. (c) Ki67-positive cells greatly reduced after BMP treatment compared with the control group. Ki67 was observed by immunofluorescence staining. (d) Cell amount presented that BMP4 suppressed the proliferation of monkey NSC-like cells. (e) The increase of cell adherent area showed that single cell became large after BMP4 treatment. (f) The length-width ratio suggested the cells became oblate from leptosomic type after BMP treatment. (g) Cells appeared shorter than control after BMP treatment. (a) and (b) Scale bar = 50 *μ*m; (c) scale bar = 25 *μ*m. Data represent the mean values ± SD; ^∗∗∗^*p* value < 0.001.

In consideration of the important functions of BMP in development, we detected the influence of BMP4 factor in NSCs. We found that BMP4 remarkably affected cell morphology (Figure 4(a)) and the cell's body became flat at high magnification (Figure 4(b)). Ki67, a marker protein of ribosomal RNA transcription, is a nuclear protein which is necessary for cellular proliferation. In previous reports, scientists adopted Ki67 to investigate cell proliferation and quiescence. We also used Ki67 to detect proliferation and quiescence of monkey NSCs. The photos showed that Ki67-positive cells obviously reduced after immunofluorescence staining in monkey NSCs (Figure 4(c)).

It has been showed that BMP4 suppressed monkey NSC proliferation (Figure 4(d)). Single cell exhibited a larger size after BMP4 treatment (Figure 4(e)). The length-width ratio suggested that cells changed into oblateness from leptosomic type after BMP treatment (Figure 4(f)). Cells appeared shorter after BMP treatment when compared with the untreated group (Figure 4(g)).

#### 3.1.5. BMP4/LIF and RA/Forskolin Suppressed Monkey NSC Proliferation

Both BMP and LIF promote the differentiation of mouse neural stem cells into mature astrocytes and other glial fibrillary acidic protein (GFAP) immunoreactive cells [36]. RA can induce human neuroblastoma cell differentiation into neuronal-like cells [37]. Forskolin, a cyclic adenosine 3′,5′-monophosphate (cAMP) activator, increases neuregulin receptors in human Schwann cells [38, 39].

**Figure 5:**
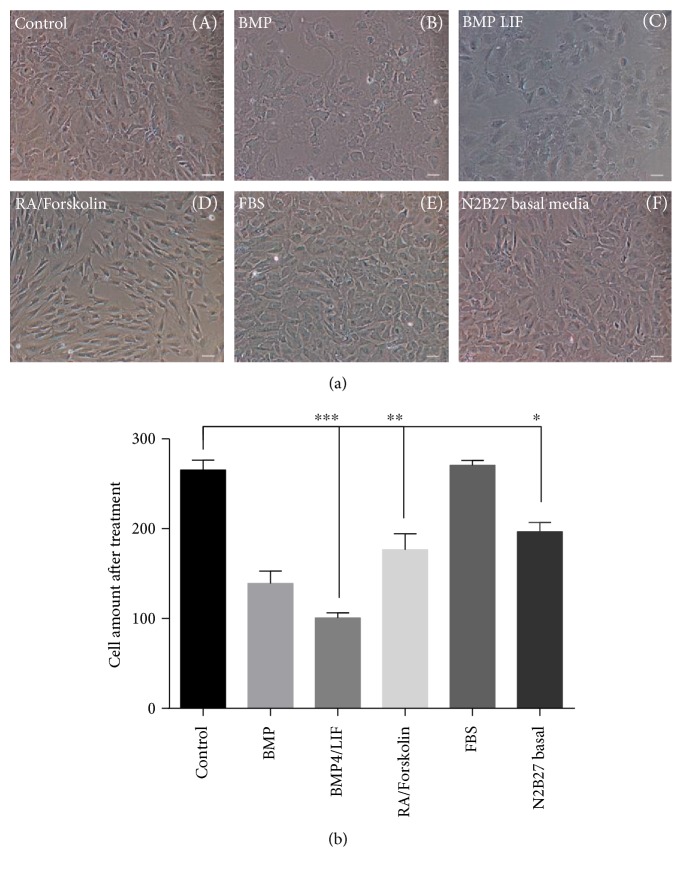
BMP4/LIF and RA/Forskolin suppress cell proliferation. (a) Monkey NSCs were treated in control medium or other rich with factors medium for 6 days. (A) Control, (B) BMP group, (C) BMP4/LIF group, (D) RA/Forskolin groups, (E) FBS group, and (F) N2B27 group. BMP4/LIF and RA/Forskolin groups showed significant difference. (b) Cell amount presented that BMP4/LIF and RA/Forskolin suppressed the proliferation of monkey NSC-like cells. Bar = 50 *μ*m. Data represent the mean values ± SD; ^∗^*p* value < 0.001; ^∗∗^*p* value < 0.01; ^∗∗∗^*p* value < 0.001.

According to the function of above factors, we used different combination of these factors testing their influence on monkey NSCs. The phenotype under different factor conditions was presented, and BMP4/LIF or RA/Forskolin dramatically changed cell morphology (Figure 5(a), A–F). BMP4/LIF (Figure 5(a), C) and RA/Forskolin (Figure 5(a), D) groups showed significant difference compared with control (Figure 5(a), A). BMP4 dramatically changed cell morphology (Figure 5(a), B) as detected before (Figure 4(b)). The changes in fetal bovine serum are not obvious within a short time (Figure 5(a), E). We also exposed monkey NSCs to spontaneous differentiation condition in order to test their differentiation abilities (Figure 5(a), F). We had observed that the neuron-like cell and astrocyte-like cell appeared in spontaneous differentiation condition (Figure 5(a), F). BMP4 and LIF seemed to promote the differentiation of neural stem cells into astrocytes (Figure 5(a), C). RA and Forskolin may advance the process of monkey NSCs differentiating into neuron (Figure 5(a), D). Cell amount suggested that BMP4/LIF and RA/Forskolin suppressed the proliferation of monkey NSCs (Figure 5(b)).

#### 3.1.6. BMP4/LIF and RA/Forskolin Promoted the Change of Cell Morphology

**Figure 6:**
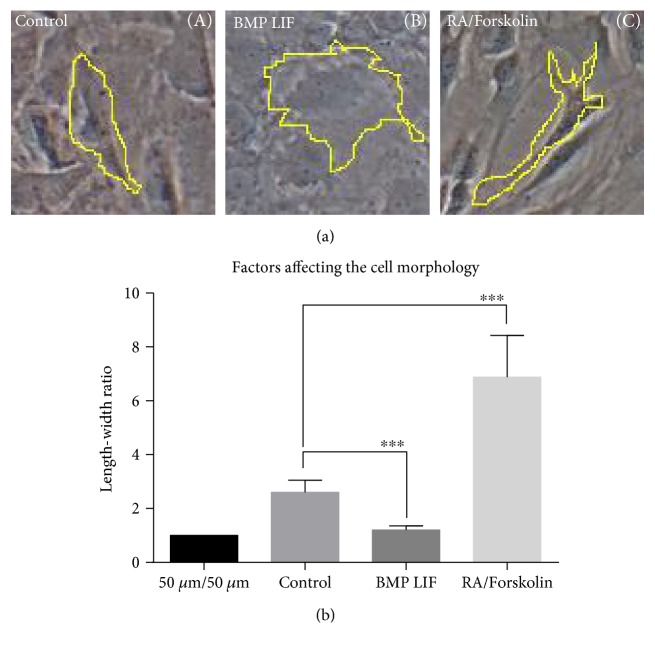
BMP4/LIF and RA/Forskolin affected the cell morphology. (a) Monkey NSCs were treated in control media or BMP4/LIF or RA/Forskolin factors media for 6 days. (A) Control; (B) BMP4/LIF group; (C) RA/Forskolin groups. The phenotype of single cells in BMP4/LIF and RA/Forskolin groups showed significant changes. (b) The length-width ratio suggested that BMP4/LIF and RA/Forskolin affected the cell morphology and may induce monkey NSC-like cells differentiation. Scale bar = 50 *μ*m. Data represent the mean values ± SD; ^∗∗∗^*p* value < 0.001.

The phenotype of single cells showed significant changes after BMP4/LIF or RA/Forskolin treatment (Figure 6(a)). BMP4 and LIF seemed to promote the differentiation of neural stem cells into astrocytes (Figure 6(a), B) compared with control (Figure 6(a), A). RA and Forskolin may advance monkey NSCs differentiating into the neuron (Figure 6(a), C). The length-width ratio suggested that BMP4/LIF had an effect on the cell morphology and may promote monkey NSC differentiation to mature astrocytes (Figure 6(b)). RA/Forskolin may promote the differentiation of neural stem cells into neurons according to the phenotype of cells and cell bodies (Figure 6(b)).

## 4. Discussion

Our study firstly demonstrated that BMP4 inhibits cell proliferation and affects cell morphology of monkey NSCs. We also discovered that combining the application of BMP4 and LIF or RA and Forskolin represses the proliferation of monkey NSCs. We also observed that BMP4 may promote quiescence of monkey NSCs. It may be essential to identify the influence of these factors to monkey NSCs, and our results may supply some helpful information to cell transplantation in practical clinical trials. Our study suggested monkey NSCs could be utilized as a useful platform for translational research. This study also may bring some theoretical support for cell therapy of human brain injury.

Brain injuries such as a traumatic injury, stroke, or other neurodegenerative disorders are life-threatening damage and leading causes of death and disability in the population worldwide, with an extensive range of symptoms and disabilities [40–42]. Brain injury causes neural cell death when the damage occurs and tissue lacks blood oxygen supply. The effective treatment for stroke is quite limited [43]. There are numerous challenges and hurdles in both academic and preclinical trials of translational stroke research [44–46].

At present, there is no particularly effective treatment for brain injuries. The capability of an adult mammalian brain to remedy the neuronal defeat which causes injury or disease is very limited [47]. Cell transplantation such as NSCs, neural progenitor cells, or mesenchymal stem cells has been considered as possible therapies for brain injury [47]. Transplantation cells aim to replace lost neurons. There is a report that embryonic neurons are transplanted into the visual cortex of adult mice; then, these grafted neurons can mature into bona fide pyramidal cells and integrate with neocortical circuits in the adult brain [48].

iPS cells can differentiate into a broad variety of neural type cells, and we may take them as an attractive donor source for autogenously neural transplantation therapies for brain injury repair [49]. However, iPS cell transplantation faces ethical barriers and risk of cancer formation. At the same time, transplanted iPS cells in vivo are difficult to produce a clear result in an extensive diversity of preclinical models for brain injury.

NSC cells sustaining neuron regeneration discovered in SGZ and SVZ are considered to be an endogenous neuroprotective device for these brain injuries. However, present strategies cannot suitably improve functional recovery after brain injury like stroke because NSCs and their microenvironment are very complex and multiple [41]. Searching for the fate-determining mechanism and studying the NSCs' performance are extremely important, including cell proliferation, migration, and differentiation.

Monkeys are the most common nonhuman primates which are employed in biomedical study. They are closer to human and suitable as a model of human disease. Moreover, monkeys such as rhesus macaque (*Macaca fascicularis*) have lower cost compared to other nonhuman primates. Neural stem and progenitor cells isolated from adult rhesus were found that these cells can differentiate into immature oligodendrocytes [31]. BDNF can support NPC proliferation and induce monkey neural progenitor differentiation into neurons, and these cells are isolated from adult cynomolgus monkeys [32]. Here, we showed detailed property analysis of monkey NSCs and we firstly investigated the inhibition effects to monkey NSCs such as BMP4, BMP4 and LIF, RA, and Forskolin.

We found BMP4 inhibited the cell proliferation and influenced cell morphology of monkey NSCs. The proliferation of cells was suppressed by application of BMP4 and LIF or RA and Forskolin. We also observed the changes of cell fate after factor treatment. Through analysis, we discovered that BMP4 alone may promote the monkey NSC quiescence. But this was just a speculation, and we needed to do more tests.

After BMP4, BMP4 and LIF, or RA and Forskolin treatment, what is the final fate of the monkey NSCs? Are the factor actions reversible? What is the impact of these factors to the NSC transplantation rate? In the following study, we will pay more attention to these questions and explore the intrinsic signaling mechanism that regulates cell proliferation, differentiation potential, and cell cycle of monkey NSCs after cytokine treatment. Identification of these mechanisms may be helpful to understand the application foundation of cell transplantation and may provide some useful information to further targeted cell therapies.

## 5. Conclusions

We firstly demonstrated the effects of BMP4, BMP4 and LIF, RA, and Forskolin on the monkey NSCs and showed detailed property analysis of monkey NSCs. We found that BMP4 inhibited the proliferation and affected monkey NSC morphology. BMP4 and LIF or RA and Forskolin suppressed proliferation of monkey NSCs. Identification of these factors' functions to monkey NSCs may be helpful to understand cell transplantation application and may provide some useful information to guide the cell therapy progress.
